# Why Do People Choose a Particular Dog? A Mixed-Methods Analysis of Factors Owners Consider Important When Acquiring a Dog, on a Convenience Sample of Austrian Pet Dog Owners

**DOI:** 10.3390/ani14182634

**Published:** 2024-09-11

**Authors:** Kata Mária Udvarhelyi-Tóth, Ivaylo B. Iotchev, Eniko Kubinyi, Borbála Turcsán

**Affiliations:** 1 Department of Ethology, ELTE Eötvös Loránd University, 1117 Budapest, Hungaryturcsan.borbala@ttk.elte.hu (B.T.); 2 MTA-ELTE Lendület “Momentum” Companion Animal Research Group, 1117 Budapest, Hungary; 3 ELTE NAP Canine Brain Group, 1117 Budapest, Hungary; 4 Clever Dog Lab, Comparative Cognition, Messerli Research Institute, University of Veterinary Medicine Vienna, Medical University of Vienna, University of Vienna, 1210 Vienna, Austria

**Keywords:** dog acquisition, pet, sporting dog, family composition, owner demographics

## Abstract

**Simple Summary:**

Choosing the right dog that fits well with an owner’s lifestyle is important for the happiness of both the dog and the owner. This study looked into why and how people in Austria choose their dogs by surveying over a thousand dog owners. Unlike past studies, we asked open-ended questions so that the owners could freely share their reasons. On average, the owners gave two to three reasons for their choice. The most common reasons included the dog’s breed, picking a dog on a whim, the dog’s abilities for work or sports, and rescuing a dog. Surprisingly, less than 1% of owners prioritized guarding abilities, and 1% considered basic traits like the dog’s sex, age, or health as key factors. If an owner picked a dog for its looks or skills, they often chose their next dog for the same reasons. Factors like the owner’s age, household composition, previous experience with dogs, and the intended role of the dog also played a part in the decision. For instance, owners with children preferred dogs that were friendly, calm, and easy to train, and they were less likely to adopt from shelters compared to those without children. Overall, our study shows the wide range of reasons that people have for choosing their dogs, which can help ensure better matches between dogs and their owners, improving the well-being of both.

**Abstract:**

Selecting a dog that is incompatible with the owner’s expectations can negatively impact both parties. Previous studies on dog acquisition have primarily focused on shelter environments, using closed-ended questions to assess hypothetical preferences. In contrast, our study employed open-ended questions with a convenience sample of Austrian dog owners (N = 1077) to retrospectively explore why the owners chose their dogs. We also examined consistency in owners’ responses and the influence of owner characteristics (age, education, household composition, previous dog experience, purpose of acquisition) on their reasons. Content analysis revealed 24 codes; the frequency of codes was 2.4/response. The most frequent codes were breed-based choice (29%), choosing on a whim, without careful consideration (24%), work/sport skills (22%), and rescuing a dog (17%). The least frequent were the age (1%), health (1%), sex (1%), and guarding skills (0.6%) of the dog. Twelve codes were consistent over time, and ten were consistent across dogs, indicating that the owners showed a consistent preference for certain traits. Except for the owner’s education level, all characteristics affected the likelihood of mentioning at least one code. Most associations were found with the presence of children in the household: owners with children preferred friendly, easily manageable, and easy-to-train dogs and were less likely to adopt or rescue compared to owners living without children. Our findings also highlight discrepancies between spontaneous (free-text) reports and responses to closed-ended questions, underscoring the importance of qualitative data in better understanding the motivations behind and the factors influencing dog acquisition.

## 1. Introduction

Dogs can play important roles in humans’ lives, not only as working animals but also as social partners [1,2]. Dog ownership can lead to numerous psychological and physical benefits for the owners [3]. However, the ability to realize these benefits often depends on the quality of the dog–owner relationship [4]. Moreover, the One Welfare approach highlights a strong link between animal welfare and human well-being [5]. Therefore, selecting a dog that is incompatible with the owner’s expectations and lifestyle can negatively impact both parties. Understanding why, how, and based on what characteristics owners acquire a dog could benefit both human and canine welfare and contribute to a better understanding of the human–dog relationship in general.

Although dog acquisition research is still relatively new, Holland’s review [6] summarizes our current understanding of why people decide to obtain a dog as a pet or a working partner (see also [7,8]), where they obtain it (e.g., [9,10]), whether they conduct research before acquiring the dog [11], why and how they select certain breeds and breed types [12,13,14,15], which behavioral and external characteristics enhance the adoption success of shelter dogs [16,17,18,19], and which characteristics the owners consider ideal for a dog [20,21]. However, previous studies also have some limitations [6]. They are limited by (1) their focus on shelter environments, (2) the assessment of owners’ preferences rather than their actual dog selections, (3) relying on closed-ended questions, (4) the lack of combining qualitative and quantitative analytical approaches, (5) the lack of study on the consistency of responses, and (6) the lack of study on the association between the owners’ acquisition choices and their demographic characteristics. In this study, our aim was to fill these gaps. Below, we detail the gaps and our proposed solutions.

1. We focused on companion dog owners who could acquire their dogs from any source, in contrast to previous studies that have been conducted in shelter environments. It is likely that, when adopting a dog from a shelter, owners may consider different traits and features compared to acquiring a dog from a breeder, for example.

2. We decided to investigate which characteristics owners considered important when deciding on a particular dog after they had acquired the dog. As most research has assessed the (prospective) owners’ preferences or intentions for certain characteristics rather than their actual selections, it remains unclear whether the final choices align with these preferences (although see [16]).

3. We turned toward open-ended questions in our survey because, although using closed-ended questions is the predominant approach in this research area, it restricts participants’ responses to a specific, predetermined set of characteristics. However, an owner’s choice of a specific dog is likely influenced by a combination of factors, such as the dog’s external and internal features, the owner’s past experiences with similar animals, their plans for the dog, their current life circumstances, and the circumstances surrounding the acquisition. Closed-ended questions typically focus on only one aspect and do not allow for a wide range of possible responses [22,23]. Offering a checklist of alternatives for preference can suggest that these are the traits that others (including the researchers) consider to be reasonable answers [24], steering participants towards the “expected” answers [25] and increasing the possibility of social desirability or conformity bias. Open-ended questions have been suggested to be more useful when asking “Why?”, particularly in memory-based questions [24] and when the question may elicit a wide variety of responses [23]. These answers are less biased by experimenter expectations and could provide more in-depth information, forming the basis of future quantitative studies. 

4. We combined qualitative data analysis with quantitative approaches. This is not without precedent, provided the sample is large enough for meaningful analyses [15,26]. First, we identified codes and themes using content analysis, and then we compared systematic differences in the frequency of the identified codes and themes across different groups. This method allowed us to compare owners in different life situations, with varying household compositions, dog-related experiences, expectations, and education, who could be expected to favor different traits when selecting an animal. However, detailed knowledge of how these factors affect dog acquisition preferences is missing. The studies closest to this direction asked dog owners about their ideal dog’s characteristics [20,21]. These studies showed that women preferred dogs that were “calm/compliant”, “sociable/healthy”, and non-aggressive, while men preferred “energetic/faithful/protective” dogs. Parents of small children described their ideal dog as less energetic and less protective [21] compared to non-parents. The training and adaptability of the dog were more important to older owners, owners living alone, and owners with more experience with dogs [20], while energetic and protective dogs were preferred by owners of multi-dog households, younger, less experienced owners, and owners without children [20,21]. However, it is important to highlight that the traits that owners select from a predefined checklist for a hypothetical “ideal” dog and the traits that they consider when making their selection could be very different (see also the stated–revealed preference gap in [16]). Moreover, by aiming to describe the ideal dog, these studies investigated only dog characteristics and, therefore, could not fully represent all the factors that may affect dog acquisition in real life. Consequently, little is known about how direct experience with similar dogs or breeds, practical reasons like knowing a reliable breeder, sentimental reasons, or unforeseen, circumstantial reasons may shape the owners’ decision to select a particular dog. 

5. We also investigated the consistency of dog owners’ responses over time by comparing their responses about the same dog approximately eight months apart. This type of reliability analysis has not been previously applied to qualitative data, making it a significant contribution to the existing research. While test–retest reliability is a standard practice in quantitative studies (e.g., [27]), its application in qualitative research is less common, albeit crucial for evaluating the consistency of subjective responses. 

6. We examined whether the owners prioritized the same characteristics when choosing a new dog, assessing the consistency of responses across different dogs. This aspect of our research aimed to determine whether dog selection behaviors reflect a stable set of preferences or whether they vary based on the specific characteristics of each dog. Our hypothesis in this respect posits that owners consistently seek the same traits in every dog, based on the assumption that factors such as the owner’s social, demographic, and individual characteristics (e.g., personality, lifestyle, family structure) influence their dog selection ([28]; see below). These characteristics are expected to remain relatively constant across different dogs, potentially leading to similar preferences in dog selection. However, the opposite (that is, owners choose each dog for different reasons) is also supported by some indirect evidence, such as the observation that dogs in multi-dog households might be acquired for distinct purposes—one for companionship and another for work or sport [29]. The traits required for a working or sporting dog may differ from those ideal for a good companion and family dog (e.g., [30]), potentially leading to differing preferences in dog selection. Furthermore, in households with an existing dog, the need for the new dog to be compatible with the old one to maintain peaceful coexistence may influence the traits deemed important for the second dog, diverging from those considered for the first. Ultimately, understanding whether owners consistently prioritize the same traits or adapt their preferences based on individual dogs will provide insights into the decision-making processes that shape dog acquisition.

All in all, while the question of what factors influence owners when acquiring dogs has received increasing attention over the last decade, there are still many gaps in the literature, particularly regarding how and why owners select a particular dog. To address these gaps, we used an open-ended, free-text question format to gather qualitative information on why and how companion dog owners selected their current dog. We then investigated whether the owner’s dog choice showed consistency over time and across different dogs. Additionally, we explored whether certain characteristics of the owners—namely, their age, education level, household composition, previous dog experience, and the purpose of acquiring the dog—influenced the factors that they considered important when choosing the animal. Addressing these questions not only contributes to a better understanding of dog owners’ decision-making processes but also provides insights that could improve dog adoption practices by tailoring recommendations to the varying preferences of different owner demographics.

## 2. Materials and Methods

### 2.1. Subjects

Our subjects were Austrian dog owners who volunteered to participate in one or more dog behavior studies at the Clever Dog Lab (CDL), initially at the University of Vienna, and later at the University of Veterinary Medicine Vienna, Austria. They agreed to be included in the “dog-owner database” of the CDL to be contacted for future studies; therefore, the majority of the respondents lived in or around Vienna. Participation in the database was voluntary, and the owners were not compensated, monetarily or otherwise. Data for this study was collected between 3 December 2010 and 8 January 2015. Over this five-year period, numerous studies were conducted at the CDL, each with different participation requirements and recruitment strategies. Studies were typically advertised through social media and the CDL website, and recruitment primarily occurred via email or phone. Owners could participate with multiple dogs, completing separate surveys for each one. We collected N = 1198 surveys in total, of which n = 1145 were unique and n = 53 were repetitions. 

### 2.2. Procedure

The owners were asked to complete an online survey to enter the dog-owner database of the CDL, and the dataset used in this study is a subset of this database. The survey typically took approximately 20 min to complete. It comprised 39 questions, primarily categorical or multiple-choice, covering various aspects:

- Demographic attributes of the owner (e.g., age, sex, education, type of residence);
- Composition of the household (e.g., number of adults, children, other dogs);
- Demographic attributes of the dog (e.g., age, sex, breed, reproductive status, origin);
- Dog-keeping practices and conditions (e.g., where the dog stays, shared activities with the owner, training activities, competitions);
- Practical information relevant to behavioral studies (e.g., owner’s contact information, food and toy motivation, health and behavioral problems, dietary restrictions of the dog).

The focal point of our study was the open-ended question “Why did you choose this dog?” (“Warum haben Sie sich für diesen Hund entschieden?”), where the respondents provided responses in a free-text format, without predefined options. There were no word limits or minimum word count criteria set for the responses. This question was positioned after the owner demographics and household composition questions but before dog-related questions that might remind the owner of factors influencing their dog choice (such as age at acquisition, sex of the dog, or origin).

### 2.3. Statistical Analyses

#### 2.3.1. Coding the Open-Text Responses

First, we categorized the types of responses based on how much detail the owners provided and how many different codes we could identify within a given response. Next, we employed content analysis [31,32,33] to identify and code the occurrence of different reasons in the free-text owner responses into narrower categories (“codes”) and broader categories (“themes”). 

**Figure 1:**
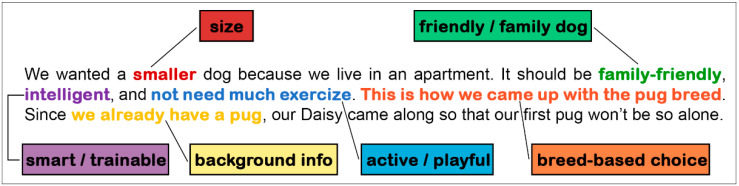
An example of how the responses were coded in the content analysis. This response contained six codes; the words or expressions identified as belonging to a given code are highlighted in different colors. The coding was carried out on the original German text. The translation into English is provided for illustrative purposes only.

**Codes:** An initial set of codes was established by the last author (B.T.) based on a pilot sample of 100 Hungarian dog owners by identifying separate segments within the raw responses that referred to different reasons and assigning them into separate categories. This coding scheme included codes such as size, work performance, and previous experience with similar breeds. The first author (K.M.U.-T.) then used this coding scheme to code the free-text German responses of the Austrian owners. The responses were manually coded using the emergent coding approach [34]. During coding, the coder assessed the presence or absence of each code on a binomial scale: 1 if the response contained any reference to that code, and 0 if it did not. The initial coding scheme was further refined and expanded whenever a new reason emerged in a response, or when a code could be subdivided into separate codes [35]. Whenever the coding scheme changed, the coder revisited previously coded responses and revised them according to the current code set and definitions. The codes were mutually exclusive, and a response could reference any number of them. We employed the representational approach [22]; that is, the coder did not search for specific keywords but aimed to understand the responder’s intended meaning and consider the context of the text [34]. Accordingly, different words or phrasings expressing the same reason were allowed. For instance, mentioning a preference for both small and large body sizes would receive a 1 for the *size* code. Likewise, preferring dogs with high or low activity levels would both be categorized under the *activity* code. An example is provided in Figure 1. 

**Themes:** Once all responses were coded and the coding scheme was finalized, we inductively grouped codes that were related to each other in their content and often mentioned together by the owners, and we labeled them as “themes”. Themes were expected to provide a more general impression of the data [35,36]. 

The outputs consisted of a series of binomial variables for each response, indicating which codes and themes were found in any given text response. As our coding method allows for subjective interpretation by the coder, it was crucial to define each code as precisely as possible. Additionally, it is important to test whether different coders detect the same codes in specific responses [31]. To assess inter-coder reliability, we utilized Cohen’s kappa on a subsample of N = 100 responses that were independently coded by the first and second authors (K.M.U.-T. and I.B.I.). This statistical measure helped us to evaluate the agreement between coders beyond what would be expected by chance.

After that, we examined the frequency of each code and theme. Codes and themes with a mentioning frequency lower than 5% of cases were discarded from the quantitative analyses, as they were too rare for reliable statistics.

#### 2.3.2. Consistency of the Responses

We assessed the test–retest reliability (repeatability) of the codes and themes that met the 5% minimum frequency threshold. We utilized Cohen’s kappa on a subsample of n = 53 responses where owners completed the questionnaire twice for the same dog (mean time between measurements ± SD = 8.0 ± 6.8 months). Aside from analyzing the consistency of each code and theme, we also investigated the consistency of the response in general by testing whether there was a correlation between the test and the retest responses in the length of the response and in the number of different codes mentioned, using Spearman correlation. 

We also investigated consistency across dogs, i.e., whether multi-dog owners’ preferences in selecting one dog were consistent with their selection of another dog. We analyzed a subsample of n = 113 owners who completed the questionnaire for at least two dogs and compared their responses between the dogs using Cohen’s kappa. In the case of owners who filled in the survey for more than two dogs, we randomly selected two for the assessment because there were not enough subjects for a three-way comparison. Similar to the test–retest analysis, we also tested the correlation in the response length and in the number of different codes mentioned between the owner’s two dogs. 

#### 2.3.3. Owner Characteristics vs. Preferences in Dog Choice

In our analysis, we included only those themes that met the 5% frequency criterion and had at least fair repeatability (Cohen’s kappa > 0.4, [37]).

We employed generalized linear models (GLMs) with a binomial distribution assumption to explore potential relationships between owners’ dog-choosing preferences and their demographic characteristics. The dependent variables in the models were the themes extracted from the owners’ responses. We conducted one model for each theme eligible for analysis based on the previously described frequency and repeatability criteria. We examined the main effects of the owner’s age (continuous), education level (middle school, technical school, college or university), presence of other adults in the household (yes, no), presence of child(ren) in the household (yes, no), presence of a dog in the household at the time of the acquisition (yes, no), previous experience with dogs (yes, no), and the role of the dog in the family (companion only, sport or work purposes). We applied backwards elimination-based model selection, removing non-significant effects from the model while also checking the AIC value at each step to ensure that removing a non-significant effect did not worsen the model’s fit. The effect sizes of the pairwise differences were estimated using odds ratios (Exp(B)). All statistical analyses were conducted using SPSS v28.

## 3. Results

### 3.1. Subjects

In total, we collected 1145 unique surveys. Among them, n = 8 were incomplete, providing no response to the target question of this study (“Why did you choose this dog?”). Additionally, in n = 5 cases, the owners described why they acquired a dog without indicating why they selected that specific animal (e.g., “We had to put the previous dog to sleep early”). These were excluded, accounting for just over 1% of the total dropout rate. However, some other responses were ineligible for inclusion due to demographic reasons (n = 55 in total), including owners under the age of 18 years (n = 12), those with no schooling experience (n = 1), individuals not identified as the primary owner of the dog (n = 18), and those who did not keep the dog as a companion (n = 24). After these exclusions, N = 1077 responses remained.

The majority of the respondents were female (88.3%), aged between 25 and 50 years, and lived in households without children (80.3%). Their dogs were predominantly acquired before their first year of age (86.8%) from breeders or private hands (79.3%). Further demographic details of the sample can be found in Table 1. 

In addition to the N = 1145 unique surveys, we also identified n = 53 repetitions (i.e., surveys filled out for the same dog twice). These entries were used solely to assess the repeatability of the owners’ responses.

### 3.2. Content Analysis

The participants’ responses exhibited a wide range in length, varying from 5 to 1787 characters (mean = 130.1, SD = 149.3, median = 83).

Based on the content analysis of the N = 1077 responses, we identified 24 codes related to why owners selected that particular dog. Inter-coder reliability exceeded 0.6 for all codes, indicating an acceptable level of agreement between coders (see Table 2). On average, the owners indicated 2.35 (SD = 1.36, min. = 1, max. = 9, median = 2) codes per response. 

We were able to distinguish four types of responses based on how extensively the owners answered and how many different codes we could identify within a given response:

- Long response with multiple different codes: In many cases, owners provided extensive descriptions of their dog’s characteristics, as well as the reasons and circumstances behind their selection. For instance, the owner with ID 115 wrote this regarding their miniature spitz: “*Standing ears, which reduce the likelihood of ear infections; long muzzle, which reduces the likelihood of respiratory problems; balanced build, which reduces the likelihood of damage to the postural apparatus; long double-layered coat, which makes the dog less sensitive to cold weather and wetness, in summer you can still shave him. So due to health reasons*” (ID115, miniature spitz). In this response, we can identify four codes: looks (general), hair, size, and health.
- Long response with few different codes: In responses like these, owners elaborated on the adoption process, yet only a few reasons were distinguishable: “*It was clear to us right from the start: if we take in a dog, it should be one that will be helped. So, we came across a couple who rescue dogs from various countries from killing stations and pass them on. That’s how we found our Benji. We saw him and just had to give him a nice home*” (ID21, mixed breed). In this response, two codes can be identified: shelter/rescue, and choosing on a whim.
- Shorter response with multiple different codes: In responses like these, owners provided brief descriptions, often using just a word or two, to convey the desirable qualities of the dog. Despite the brevity, multiple reasons were distinguishable in the responses. For example: “*active, friendly, size*” (ID240, Hungarian vizsla). The codes found in this response are active/playful, friendly/family dog, and size.
- Shorter response with fewer different codes: In responses like these, owners provided brief descriptions, often with just a single word, to describe the adoption of the dog. Even in responses with multiple words, only one or two reasons were distinguishable: “*Working dog*” (ID229, border collie). In this response, only one code is found: work/sport skills.

#### 3.2.1. Codes 

In the following subsection, we provide a description of the codes that we identified in the owners’ responses. To facilitate understanding of the coding scheme, we provide examples of various words and phrases that the owners used in relation to each code. After the name of the code, we present the shortened version name that we will use hereafter, as well as the frequency (appearance/all responses).

1. Preference, love, and fascination of the breed (*breed-based choice*, 29.43%): In nearly one-third of the responses, the owners indicated that they selected the breed and not the individual, either by stating that they liked the breed, or by mentioning the name of the breed in the response. Examples: “*Because I like this breed*” (ID281, American Staffordshire terrier); “*The breed best met our requirements.*” (ID73, French bulldog); “*I fell in love with Border Collies in general on a winter holiday. When the opportunity arose to get a puppy, we decided on a female* […]” (ID736, border collie); “*A Golden Retriever has always been my dream* […]” (ID824, golden retriever).
2. The look of the animal (*looks (general)*, 10.03%): In some instances, owners solely referenced the appearance or overall look of the dog, while in others, they emphasized the animal’s attractiveness. Owners typically mentioned the animal’s appearance (“appearance”), perhaps the body structure (“body structure”), or used terms denoting beauty (“beautiful”, “pretty”, “gorgeous”). Some owners appreciated their pets for resembling another breed but being more practical for their lifestyle. Examples: “*In love with looks*” (ID254, mixed breed); “[…] *because he is such a beautiful dog*.” (ID912, border collie); “*looks exactly like a Doberman, only it’s smaller*” (ID501, German pinscher).
3. The coat of the animal (*hair*, 3.62%): Some owners specifically mentioned the color (“white”, “colorful coloring”, “funny pattern”, etc.), the length (“long haired”, “short haired”, “not too long or not too short”, etc.), or used words to describe the texture of the coat (“fluffy”, “bushy”). Others noted the lack of shedding as an important aspect (“does not shed hair”) and the ease of care for the type of hair (“easy to clean”, “easy to groom”). Examples: “*Does not shed as much hair*” (ID439, Tibetan terrier); “*She caught our eye* […] *certainly because of her funny coloring.*” (ID50, mixed breed); “*I always wanted* […] *light-colored coat that wasn’t too long or too short*” (ID749, mixed breed).
4. The size of the animal (*size,* 10.77%): Owners often emphasized their preference for a dog of a specific size (“small”, “medium”, or “large”). Some considered the size of their home or the dog’s portability (“optimal apartment sized dog”, “easy to take anywhere”), while others considered future joint activities (“optimal size for rescue dog work”). Examples: “*I wanted a working dog, medium-sized.*” (ID265, border collie); “*good size for a city apartment in Vienna*” (ID418, standard schnauzer); “*can be taken almost anywhere due to its size*” (ID581, Chihuahua).
5. Being a shelter dog, adopted or rescued, or a saved dog (*shelter/rescue*, 17.08%): Many owners expressed a specific desire to adopt or rescue a dog from a shelter. Some mentioned fostering the dog before adoption, while others sought dogs that had been in shelters for a long time. Some owners rescued their pets from the streets, emphasizing phrases like “found on the highway” or “found on the street”. It was important to some owners that the dog came from another country or from a killing station (phrase: rescued from killing stations). Examples: “*I wanted to save her*” (ID722, mixed breed); “*we adopted her*” (ID359, cocker spaniel); “*I wanted to give a* […] *dog that had been in the shelter for a long time a place to live.*” (ID415, mixed breed).
6. Lineage, breeder, or the parentage of the animal (*pedigree*, 4.55%): Some owners emphasized the lineage or parentage of the dog (“pairing of parents”) or its good pedigree using the phrases “good bloodline”, “good parents of the dog”, and the “pedigree of the parents”. They considered factors such as the reputation of the breeder (“breeder friend of mine”, “known the breeder”), as well as being from a good background (“from a good place”, “trusted breeding”). Familiarity or positive past experiences with the breeder also influenced their choice (“from the same breeder”). Examples: “*We know the mother and father of our bitch.*” (ID836, Labrador retriever); “*I bought her* […] *from the same breeder where we bought our Pumi bitch*” (ID710, mudi).
7. The health of the animal (*health*, 1.39%): Some owners prioritized finding a dog that was in good health and free of breed-specific diseases. They described specific health criteria (“too small for HD, too large for patellar luxation”), or simply emphasized the importance of the dog being “healthy”, “not sick”, “no health complaints”, or “free from hereditary diseases”. Examples: “*With the hope of obtaining a healthy dog*” (ID44, harzer fuchs); “*breed with no typical breed complaints to speak of*” (ID24, Parson Russell terrier).
8. The age of the animal (*age*, 1.49%): Some owners mentioned the age category of the dog they were looking for, such as preferring a “young” or “old” dog. In some cases, age was mentioned as a reason on its own (“age”). Additionally, some owners considered the expected lifespan of the individual dog (“long-lived”). “*He* […] *was the same age as Paul*.” (ID15, Irish wolfhound); “*Breed, age*” (ID157, Labrador retriever).
9. The sex of the animal (*sex,* 1.30%): Many owners emphasized their preference for a specific sex when selecting their dog (“a male dog”). Some casually mentioned the sex of the individual dog, while others considered the neutering status as an important factor (“intact”, “neutered”). Examples: “*He was the only male in the litter.*” (ID411, mixed breed); “*we wanted a bitch from this litter*” (ID914, border collie).
10. Calmness (*calm*, 2.51%): Some owners sought dogs with calm temperaments, describing this behavior as “cozy nature” or “serenity”. Others mentioned qualities like being “restrained” and “not hectic”. Examples: “*because this dog is a calm representative of her breed*” (ID206, Australian shepherd); “*After a Doberman, a calmer dog was sought* […]”. (ID784, Labrador retriever).
11. Friendliness, low aggression, being a family dog (*friendly/family dog*, 15.23%): Owners expressed a desire for a dog that would be friendly and well-behaved around people, including children and strangers. Terms like “friendly”, “dear”, “nice”, and “affectionate” were used. Alongside the term “family dog”, many emphasized kindness and sociability towards people (“people-oriented”, “friendliness towards people”, “good social behavior and people-friendly”). Many emphasized the importance of the dog being a good family companion, exhibiting kindness, sociability towards people, and being child-friendly (“loves children”, “very child-friendly”, “suitable for children”, “kind with children”) and patient (“patient”). Examples: “*She bribed me with her friendliness towards people.*” (ID269, mixed breed); “*This breed is people-friendly and likes children*.” (ID516, Bernese mountain dog); “[…] *of all puppies, this dog was the best suited as a family dog.*” (ID485, Appenzeller mountain dog).
12. Docility, easily manageable character (*docile/manageable*, 5.48%): Owners valued dogs with an easy-to-handle temperament, often described as docile, good-natured, and adaptable. Terms like “docile”, “good-natured”, “easy tempered”, and “adaptable” were also used to describe the code. In addition to “manageable” or “easy to manage”, the terms “easy to handle”, “trouble-free”, “easy”, “good for beginners”, and “not difficult” were used to express manageability. Examples: “*A mix of trusting and adaptable breeds*” (ID764, mixed breed); “*I wanted a dog that would fit into everyday life without any problems*” (ID461, golden retriever).
13. Working and/or sporting skills (*work/sport skills*, 21.82%): Owners sought dogs specifically for work or sports activities. Aside from generally mentioning “sporty” or “working dog”, the owners used a wide variety of terms to describe the characteristics of a dog that would be suitable for such activities in the future. They looked for dogs with characteristics suitable for various tasks, described as “carry out jobs”, “willing to work”, or “eagerness to work” to describe the dog’s suitability for work, or even described future activities like “guide dog training”. Those looking for a sporting dog often wrote that they were looking for a “dog for sports”, “suitable for sports”, or described the exact sporting activity as “agility”, “obedience”, “mantrailing”, etc. Examples: “*I would like to work with him.*” (ID229, border collie); “[…] *deliberately chose a dog for sporting activities such as agility, dog racing*” (ID647, mixed dog); “*Dog sports partner*” (ID304, border collie).
14. Cleverness and trainability (*smart/trainable*, 13.93%): Owners desired dogs that were intelligent, easy to train, and obedient. Characteristics associated with this code included terms like “clever”, “intelligent”, “easy to train”, “eagerness to learn”, “training possibilities”, “motivated”, and “enjoys learning”. Examples: “*Willing to work, will to please*” (ID661, border collie); “*He is very sensitive, but always good-natured, active and, above all, extremely eager to learn.*” (ID302, bearded collie); “*Because I wanted, above all, a smart, intelligent all-rounder* […]” (ID38, Australian shepherd).
15. Active, energetic, agile nature, being playful and boisterous (*active/playful*, 16.99%): Adjectives related to activity included “agile”, “athletic”, “fast”, and “lively”. The dog’s playful behavior was usually described by the words “playful” or “cheerful”. Examples: “*I liked the dog’s energy* […]” (ID437, mixed breed); “*Very motivated, active.*” (ID248, Australian shepherd); “*I wanted a lively dog*” (ID635, malinois).
16. The character, temperament, and personality of the animal (*character (global)*, 15.69%): Owners referenced the overall character, temperament, or personality of the dog using terms like “personality”, “nature”, or “temperament”, without specifying particular traits. Examples: “*Liked his character*” (ID417, mixed breed); “*character, appearance*” (ID554, mixed breed).
17. Loyalty (*loyalty*, 1.95%): Some owners referred to the dependence or loyalty of the dog, mostly using the words “loyal” and “faithful”. Examples: “*They are extremely loyal to their owners*” (ID272, mixed breed); “*Wanted a dog with a close bond* […]” (ID282, Australian shepherd).
18. Guarding skills (*guarding*, 0.56%): Owners mentioned characteristics related to the dog’s ability to guard the house or provide security. Terms like “guardian”, “vigilant”, or “watchdog” were used, possibly referring to the animal’s “protective instinct” and “sense of security”. Examples: “*I wanted a big, intelligent, but not crazy guard dog*” (ID888, Dutch shepherd); “[…] *alert dog*” (ID756, mixed breed).
19. Being stubborn, headstrong, a challenge (*stubborn*, 3.81%): Responses indicating that the dog was stubborn, independent, or posed a challenge for the owner were included in this code. Terms like “stubborn”, “naughty”, “strong-willed”, “headstrong”, or “cheeky” were used to describe the dog’s obstinacy or strong will. Examples: “*I find stubbornness very attractive*.” (ID723, Parson Russell terrier); “*The Puli is a very independent dog breed*.” ID484, puli).
20. Having direct experience with similar individuals or same breeds (*background information*, 7.89%): Owners sometimes mentioned that they relied on their previous experiences with similar breeds, breed types, or individuals when selecting their new dog. This included mentioning growing up with or owning another individual from the same breed/type, or knowing a similar dog (e.g., of the neighbor). Some owners mentioned that they knew the parents of this dog. Examples: “*I grew up with a long-haired collie and then with border collies*” (ID748, border collie); “*My parents had such a dog*” (ID542, Airedale terrier).
21. Sentimental, reminiscent responses (*sentimental reasons*, 9.38%): Responses in this code indicate that the owner’s selection of the dog was influenced by positive childhood memories, favorite books or movies, or personal beliefs. Expressions such as “a wish since childhood”, “is my dog of fate”, and “long-awaited wish” were commonly used to convey this sentiment. Examples: “*A childhood dream*” (ID600, German shepherd); “[…] *I now think that when I was looking for a dog, I remembered my childhood dog* […]” (ID819, mixed breed).
22. Unplanned, accident, falling in love (*choosing on a whim*, 23.96%): Responses in this code indicate that the owner’s selection of the dog was based on chance or an emotional connection. Expressions such as “coincidence”, “just so happened”, “by accident”, “dog’s choice”, “fell in love”, “liked it”, or “sympathetic” were commonly used to convey this sentiment. Examples: “*Love at first sight*” (ID844, mixed breed), “*that was a coincidence*” (ID402, mixed breed); “*I saw her in pictures and immediately decided that she was a good fit for us.*” (ID722, mixed breed); “*born—seen—in love*” (ID474, border collie).
23. Mentioning that somebody else also had a say in the selection (*chosen by other*, 8.17%): Responses in this code indicate that the owner’s decision to select the dog was influenced by another person. This could be their child, partner, or another individual. Expressions such as “my son brought”, “wish of the daughter”, “a wish from my wife”, “my husband fell in love”, “my father chose him for me”, or “was my foster” were commonly used to convey this influence. Examples: “*The decision was not made by me, but rather by my partner.*” (ID680, mixed breed); “*My previous one died unexpectedly, and my partner at the time immediately started looking for a new dog. We chose him together.*” (ID51, mixed breed)
24. Unique behaviors (*unique behaviors*, 4.18%): This code encompasses behavioral characteristics mentioned by only one or two respondents, which were not categorized separately due to their limited occurrence. Traits such as communication skills or being funny, happy, or sensitive fall under this category.

Finally, there were a few responses that included unique reasons not fitting into any of the previously mentioned codes, yet were too rare to form separate codes. Examples include the rarity of the breed (two mentions), the low costs of keeping the animal (two mentions), and compatibility with cats (one mention).

#### 3.2.2. Themes

We identified five broad themes inductively from the codes that were related to each other in their content [35]. The themes were also scored on a binomial scale, receiving a score of 1 if the response contained any mention of any code grouped into that theme and 0 if none of the codes were mentioned. Below, we present these themes:

1. *Appearance* (20.33%): included all three codes related to appearance, i.e., *looks (general)*, *hair*, and *size*.
2. *Origin* (21.54%): included the two codes concerning the dog’s origin, namely, *shelter/rescue* and *pedigree*.
3. *Demographics* (3.99%): included the three codes (*health*, *sex*, *age*) related to the basic, demographic characteristics of the dog.
4. *Friendly/manageable* (19.31%): we merged the three codes associated with the dog’s amicable, easy-to-handle disposition (*calm*, *friendly/family dog*, and *docile/manageable*) into this theme.
5. *Sport* (36. 86%): we grouped three codes related to the dog’s suitability for sports or work into this theme [38]: *work/sport skills*, *smart/trainable*, and *active/playful*.

**Figure 2:**
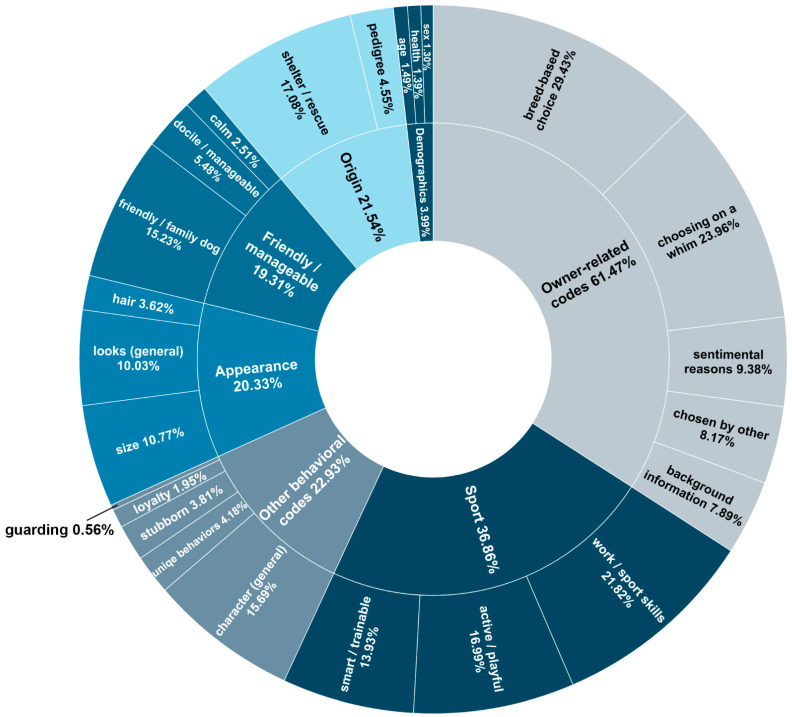
Distribution of the codes, themes, and miscellaneous code groups identified in the thematic analyses of N = 1077 free-text responses. The inner circles represent the themes, and the corresponding outer circle slices represent the codes within that theme. On average, owners mentioned 2.4 reasons in each response; therefore, the sum of the segments in the pie chart exceeds 100%.

For practical reasons, those codes that did not fit any theme were grouped into two groups (*other behavioral codes* and *owner-related codes*). The definitions and frequencies of the codes, themes, and remaining code groups, along with the results of the reliability and consistency analyses, are presented in Table 2. A visual representation of the distribution of the codes, themes, and theme groups can be found in Figure 2.

**Table 2:** Descriptions, frequency (appearance/all responses), and reliability assessments of all codes and themes (bold), were created based on the free-text responses of the owners (N = 1077). Inter-coder reliability (n = 100), test–retest reliability (n = 53), and consistency across dogs (n = 113) were calculated using Cohen’s kappa. The latter two were calculated only for codes and themes with a frequency higher than 5%. The decision regarding the inclusion in the GLM analyses and the reasons for exclusion are presented in the last column of the table.

Codes and Themes		Description	Frequency	Inter-Coder Reliability	Test–Retest Reliability	Consistency Across Dogs	Included in the GLM Analyses?
**Appearance theme**		One, if any, from looks (general), hair, or size was mentioned	20.30%	-	0.733	0.580	Included in the analyses
	Looks (general)	Any reference to the whole appearance of the dog without mentioning specifics (e.g., “looks” or “beauty”)	10.03%	0.928	0.151	0.500	Low repeatability, analyzed only as part of the theme
	Hair	Any reference to the fur or coat of the dog	3.62%	1.000			Low frequency, analyzed only as part of the theme
	Size	Any reference to the size of the dog	10.77%	1.000	0.737	0.698	Analyzed both as part of the theme and on its own
**Origin theme**		Either shelter/rescue or pedigree was mentioned	21.54%	-	0.600	0.274	Included in the analyses
	Shelter/rescue	Mentioning that they rescued the dog, adopted it out of pity, or adopted it from a shelter or rescue organization	17.08%	0.765	0.612	0.251	Analyzed both as part of the theme and on its own
	Pedigree	Mentioning the importance of the dog’s pedigree (or the lack of it), or knowing the breeder	4.55%	0.656			Low frequency, analyzed only as part of the theme
**Demographics theme**		One, if any, from health, age, or sex was mentioned	3.99%	-			Excluded due to low frequency
	Health	Any reference to the health of the dog	1.39%	0.789			Low frequency, analyzed only as part of the theme
	Age	Any reference to the age of the dog	1.49%	0.789			Low frequency, analyzed only as part of the theme
	Sex	Mentioning specifically that they wanted a male or female dog	1.30%	0.795			Low frequency, analyzed only as part of the theme
**Friendly/manageable theme**		One, if any, from friendly/family dog, docile/manageable, or calm was mentioned	19.31%	-	0.625	0.360	Included in the analyses
	Calm	Any reference to the calmness of the dog	2.51%	1.000			Low frequency, analyzed only as part of the theme
	Friendly/family dog	Any reference to the friendliness, lack of aggression, or family- or child-compatibility of the dog	15.23%	0.710	0.731	0.333	Analyzed both as part of the theme and on its own
	Docile/manageable	Any reference to the docility or easily manageable nature of the dog	5.48%	0.651	0.026	0.263	Low repeatability, analyzed only as part of the theme
**Sport theme**		One, if any, from work/sport skills, smart/trainable, or active/playful was mentioned	36.86%	-	0.801	0.409	Included in the analyses
	Work/sport skills	Any reference to working or sporting skills of any type (e.g., agility, herding, hunting, therapy)	21.82%	0.727	0.612	0.478	Analyzed both as part of the theme and on its own
	Smart/trainable	Any reference to the smartness, trainability, or obedience of the dog	13.93%	0.926	0.470	0.483	Analyzed both as part of the theme and on its own
	Active/playful	Any reference to the activity level or playfulness of the dog	16.99%	0.759	0.394	0.453	Low repeatability, analyzed only as part of the theme
**Other behavioral codes**		Miscellaneous behaviors that did not form a theme	22.93%				
	Character (general)	Any reference to the whole character or personality of the dog (e.g., “nature”, or “temperament”), without mentioning specifics	15.69%	0.687	0.009	0.076	Excluded due to low repeatability
	Loyalty	Any reference to the clinginess, dependence, or loyalty of the dog	1.95%	1.000			Excluded due to low frequency
	Guarding	Any reference to the dog’s house-guarding ability (also included if the dog provides safety)	0.56%	1.000			Excluded due to low frequency
	Stubborn	Any reference to the personality of the dog (also included if the owner wanted a challenge with the choice)	3.81%	0.795			Excluded due to low frequency
	Unique behaviors	A collective code including all and only those behavioral traits that were mentioned only once or twice in the dataset and, thus, have no code of their own	4.18%	0.729			Excluded due to low frequency
**Owner-related codes**		Miscellaneous owner-related codes that did not form a theme	61.47%				
	Breed-based choice	Mentioning the breed by name or indicating that they chose the breed specifically	29.43%	0.735	0.498	0.524	Included in the analyses
	Background information	Any reference to previous experiences with the breed, the breed type, or the parent(s) of the dog	7.89%	0.852	0.696	0.425	Included in the analyses
	Choosing on a whim	Just fancying that particular dog (typically include expressions like “I fell in love”, “the dog chose me”, “just so happened”)	23.96%	0.628	0.695	0.367	Included in the analyses
	Sentimental reasons	Any mention of sentimental reasons (e.g., selecting the dog because of good childhood memories, favorite books or movies, etc.)	9.38%	0.936	0.340	0.498	Excluded due to low repeatability
	Chosen by other	Mentioning that another person took part in selecting the dog	8.17%	0.729	0.039	0.071	Excluded due to low repeatability

### 3.3. Consistency of the Responses

In the consistency analyses, we included only codes and themes with a frequency above 5%, as codes rarer than that had insufficient frequency to be analyzed statistically. Of the five themes, one (*demographics*) did not meet this criterion, while 10 of the 24 codes did not. Table 2 shows the results of the consistency analyses for all eligible codes.

#### 3.3.1. Test–Retest Reliability (Repeatability)

All four eligible themes and eight of the codes exhibited at least fair repeatability (kappa > 0.4, [37]). The remaining six codes—namely, *looks (general)*, *docile/manageable*, *active/playful*, *character (general)*, *sentimental reasons*, and *chosen by other*—failed to demonstrate repeatability (Table 2).

#### 3.3.2. Consistency across Dogs

To investigate whether the owners consistently chose individuals based on similar traits across their multiple dogs, we compared the responses for dogs from the same owner. Among the four themes and fourteen codes with a frequency > 5%, ten exhibited at least fair consistency across dogs (kappa > 0.4, Table 2): the *appearance* theme, along with its two codes *(looks (general)* and *size*), the *sport* theme and all three of its codes (*work/sport skills*, *smart/trainable*, and *active/playful*), and from the owner-related codes, the *breed-based choice*, *background information*, and *sentimental reasons*.

Another noteworthy finding is the positive correlation observed in both the test–retest and multi-dog datasets regarding the length of the responses between the two surveys. A weaker association was noted in the number of different codes mentioned in the response (Spearman correlation, N = 53, ρ = 0.376, *p* = 0.006 for the repeated assessment of the same dog; N = 113, ρ = 0.322, *p* < 0.001 for multiple dogs), while a moderate correlation was found in the number of characters in the response (Spearman correlation, N = 53, ρ = 0.499, *p* < 0.001 for the repeated assessment of the same dog; N = 113, ρ = 0.588, *p* < 0.001 for multiple dogs).

### 3.4. Owner Characteristics vs. Preferences in Dog Choice

In these analyses, we retained only codes with a frequency > 5% and at least fair repeatability (kappa > 0.4, [37]). Four themes met this criterion, along with eight codes (Table 2). Among the codes, three were unique, and five were included in the themes. First, we conducted one model for the four themes (*appearance*, *origin*, *friendly/manageable*, *sport*) and the three unique codes (*breed-based choice*, *background information*, and *choosing on a whim*). 

Next, we conducted a separate model for each of the five codes that were included in the themes but also met both the frequency and reliability criteria individually. These codes were *size*, *shelter/rescue*, *friendly/family dog*, *work/sport skills*, and *smart/trainable*. This approach aimed to provide a deeper understanding of the results obtained from the theme models. A summary and statistical details of the most parsimonious model for each dependent variable are provided in Table 3.

In the case of the *appearance* theme, only previous experience with dogs had a significant effect: owners who had not owned a dog before were more likely to report that the appearance of the animal was a decisive factor in their choice compared to those who had owned a dog before (*p* < 0.001). For the single code within this theme that was eligible for individual analysis, *size*, we observed the same result (*p* < 0.001). This suggests that size is the primary factor that inexperienced owners consider important.

For the *origin* theme, younger owners (*p* = 0.025), owners without children in the household (*p* = 0.014), and owners who already had a dog at home (*p* = 0.034) more often mentioned the source of the dog as an important factor compared to older owners living with children and those without a dog when the focal dog was selected. Additionally, owners who kept their dogs solely as companions were more likely to emphasize the origin of their pets (*p* = 0.008). Among the codes in this theme, only the *shelter/rescue dog* code was eligible for individual analysis. For this code, we found similar differences as for the theme in terms of owner age (*p* = 0.016), presence of children in the household (*p* = 0.018), and the role of the dog (*p* < 0.001). This suggests that younger owners without children and those keeping dogs solely for companionship preferred acquiring dogs from shelters and rescue organizations over other sources. However, unlike the theme, no difference was found in the *shelter/rescue dog* code between owners who already had a dog at home and those who did not, implying that owners with dogs consider it more important to acquire their new dog from a known genetic line or a breeder. Alternatively, it is also possible that already having a dog in the household could hinder the adoption process, especially if the current dog has behavioral issues.

Regarding the *friendly/manageable* theme, owners living with children mentioned traits in this theme more often than owners with no children in the household (*p* = 0.009). Similarly, owners who did not have another dog when they acquired the focal animal showed a preference for this theme compared to those who already had another dog at the time of the focal dog’s arrival (*p* = 0.002). Analysis of the eligible code in this theme (*friendly/family dog*) only partially agreed with the results of the theme. Owners living with children and those who did not have a dog at the time of acquisition mentioned the friendliness and family-compatibility of the dog as their basis for selection more often (*p* = 0.001 and *p* = 0.024, respectively). Additionally, previous experience with dogs and owner age affected this code: younger owners and those who had had dogs before the current one mentioned their wish for a friendly family dog more often (*p* = 0.015 and *p* = 0.023, respectively).

In the case of the *sport* theme, owners who had had dogs in the past (*p* = 0.016) and owners who kept their dogs for work or sport purposes (*p* < 0.001) mentioned traits related to this theme more often. Among the codes that made up this theme, two (*smart/trainable* and *work/sport skills*) were eligible for individual analysis. For the *smart/trainable* code, similar to the theme, keeping the dog for working and sport purposes (*p* < 0.001) increased the likelihood of mentioning the trainability or smartness of the animal among the reasons for selection. Contrary to the theme, owners with other adults (*p* = 0.021) and children (*p* = 0.005) in the household mentioned smartness or trainability more often than owners living alone and without children, while already having a dog in the household decreased the likelihood of mentioning such traits (*p* = 0.018). Regarding the *work/sport skills* code, keeping the dog for working and sports purposes showed a similar increase in the likelihood of mentioning these traits as for the *sport* theme (*p* < 0.001). However, similar to the *smart/trainable* code, owners living with children also more frequently mentioned the pet’s working or sporting potential among the reasons for selection (*p* = 0.035). Meanwhile, contrary to the *smart/trainable* code, already having a dog in the household when the focal dog arrived increased the likelihood of mentioning this trait (*p* < 0.001).

Selecting a breed instead of an individual (i.e., *breed-based choice*) was related significantly only to the role of the dog (*p* < 0.001): owners who kept their dogs for work or sport purposes mentioned choosing a breed more often than owners who kept their dogs only for companionship. 

Mentioning *background information* as a reason for choosing a particular dog (i.e., having owned or having direct experience with similar breeds, breed types, or individuals) was significantly related to owner age (*p* = 0.009) and having past dog-related experiences in general (*p* = 0.014). Older owners and those with previous dog experiences were more likely to mention these reasons than those without such experiences. Finally, the *choosing on a whim* code was related to two characteristics of the owner’s household composition: people who lived without other adults or other dogs in the household (*p* = 0.006 and *p* = 0.003, respectively) were more likely to choose a dog without careful consideration.

## 4. Discussion

Our main goal in this study was to gather information about the characteristics that people living in a Western European country find important when choosing a dog. We used a free-text question format to gather qualitative information on why and how pet dog owners in Austria selected their current dog, investigated whether the owners’ dog choices showed consistency over time and across different dogs, and investigated the possible influence of the owners’ family composition, previous experiences with dogs, and the purpose of acquiring the dog on their decision.

### 4.1. Codes and Themes

We identified 24 codes in the owners’ responses, with an average of 2.4 different codes per response. Most codes focused on the physical, behavioral, or demographic characteristics of the future dog, and the majority of the responses included at least one of these codes. However, many responses also included codes that were not directly related to the dog, such as the owners’ desire to help or save a dog or their sentimental attachment to a specific type of dog. This indicates that dog-choice behavior is influenced by a combination of different factors. Unlike previous studies that used closed-ended questions with predefined response options, we used open-ended questions, allowing for free-text responses. This methodological difference means that care should be taken when comparing our results with those from studies using closed-ended questions, as open- and closed-end responses may rely on different cognitive processes [24,39]. Free-text responses enable participants to provide detailed responses with greater diversity in opinions [23], often resulting in a larger pool of themes but also a higher rate of rare themes [40]. This larger pool of themes and the high rate of rare options were evident in our dataset, where only 14 out of the initial 24 codes were mentioned in at least 5% of the responses (the minimum criterion of eligibility that we set for the analyses). However, we identified some factors that had not been captured by previous research. For instance, sentimental reasons, indicated in nearly 10% of our responses, have not been considered as a potential factor in choosing a dog in previous studies. Similarly, previous experience with dogs was usually considered only as an explanatory variable (i.e., an expected cause for a potential difference) rather than a deciding factor. However, in our sample, nearly 8% of the responses referenced previous experience with similar individuals or breeds.

Additionally, open-ended questions allow for spontaneous responses, and studies suggest that there are differences between what people think they should consider important when given a list of options and what they spontaneously report (e.g., [22]). Supporting this, in our study, less than 1% of the owners spontaneously indicated that the dog’s guarding ability was an important factor in their decision. In contrast, in the study by Cohen and Todd [16], where prospective adopters from a shelter were asked to indicate their preference for 13 predefined behavioral and physical traits for dogs, 66% of the prospective owners indicated a preference for protection.

However, open-ended questions have their own disadvantages, even aside from their more complicated and time-consuming data-cleaning procedures. Chief among these is the risk of unintentionally leaving out traits that are intuitively obvious to the respondent. This bias might explain the extremely low (<2%) frequency of mentions of basic dog characteristics such as sex, age, and health status. This is notable given that, in a study asking about the most important characteristics of an ideal dog, being physically healthy was the third most important out of 44 traits [21]. Alternatively, these omissions could reflect recall bias, highlighting a difference between our method and those of previous studies. In our research, we asked owners why they chose their dog after they had acquired it, whereas the majority of other studies assessed hypothetical preferences or the intentions of owners to select dogs with certain characteristics. Although we expected owners to recall the factors influencing their decisions somewhat accurately, it is likely that they only remembered the most important, decisive factors or those that were still somewhat relevant. Owners may not have listed certain originally important factors because, while these factors influenced their decision at the time of selection, they lost significance later. This might explain why only 20% of our owners mentioned any type of appearance traits in their responses, contrary to the much higher rates found in other studies (e.g., 75% in [18]). 

It should also be noted that, aside from the methodological differences described above, potential differences in the sampled owner population could partially account for discrepancies in the descriptives. For example, the above-cited studies investigated only owners who wanted to adopt dogs from shelters, whereas our study assessed a general pet dog population. Finally, it is also important to acknowledge that, unlike previous studies that focused on the ideals and preferences of potential owners, our research assessed the actual choices of the owners, which are often influenced by practical factors. For example, if participants encountered difficulties in acquiring dogs that matched their ideal criteria, then they may have adjusted their preferences based on availability. Future research could benefit from exploring both the aspirational and practical aspects of dog acquisition, combining studies on preferences with (retrospective) analyses of actual acquisition experiences to better understand how practical challenges shape the final choice of a dog. Nevertheless, some descriptives were similar to those found in other studies, such as our 24% of dogs being chosen on a whim compared to the approximately 25% ratio of owners choosing their dogs without careful consideration [6].

### 4.2. Consistency

Momentary states and situational constraints like forgetfulness, lack of time, or lack of interest in answering the question in depth could also have affected the code frequencies in our study. This warranted investigating the consistency of the owners’ responses over time and across dogs. 

To this end, we analyzed double entries, where an owner filled out the questionnaire for the same dog twice, with an average interval of 8 months between the first and second administration. Of the 18 themes and codes mentioned in at least 5% of the total responses, 12 were at least fairly consistent. Two of the codes that were not repeatable were generally the least specific, i.e., *looks (general)* and *character (general)*, where the owners only indicated that the appearance or behavior of the dog was a basis for their choice, without further specification. The low consistency of these codes could be explained by owners providing more specific responses on subsequent occasions. *Docility*/*manageable* was just above the 5% threshold; it is possible that its low prevalence caused its low consistency. For two other codes (*chosen by other* and *sentimental reasons*), a possible reason for the lack of temporal consistency could be that the owners may not have thought that these factors were as relevant to their answers as dog-related factors, so they may have been more likely to omit them randomly. This finding suggests that factors unrelated to the dog itself may be underreported or inconsistently recalled by owners, indicating a potential bias in studies relying on self-reported data, and highlighting the need for more robust methods to accurately capture all relevant decision-making factors in future research. Finally, while the *active/playful* code was close to the kappa > 0.4 minimum threshold, it could not be considered reliable over time (interestingly, however, it was fairly consistent across different dogs). In this case, recall bias might be the most likely explanation: the dog’s activity level was likely an important characteristic when selecting the animal, but it became less significant later in life, especially if the dog’s activity level adapted to the owner’s needs. 

Our results also showed that the owners’ preferences for dogs were consistent across different dogs in approximately half (10 out of 18) of the themes and codes included in the analysis. Traits related to appearance and sport, or work skills were consistent, while reasons related to the friendliness and origin of the dog were not. The owners were also fairly consistent in considering previous experiences and background information, preference for the breed, and sentimental reasons when choosing multiple dogs. However, codes like *chosen by other* and *choosing the dog on a whim* were characteristic only for one dog. Overall, these results suggest that owners do have a general preference for physical and behavioral characteristics when choosing their dogs, indicating that they often select dogs based on similar traits. This seems to contradict the idea that each dog is chosen based on different characteristics because they play different roles in the owner’s life [29]. Instead, it supports the notion that the psychological mechanisms behind dog choice resemble those of preferential partnership [28]. However, it should also be noted that the consistency across dogs was relatively low. Unlike repeatability, where most of the codes that passed the criteria had a good level of reliability (kappa > 0.6), only the *size* code reached that level of agreement in consistency across dogs. We also found a positive correlation in the response length between the two surveys from the same owner. This suggests that owners have their own style of responding, with some preferring longer, detailed responses and others giving short, to-the-point responses. Moreover, it shows that momentary and situational constraints did not play a large role in determining how the owners answered the question.

### 4.3. Owner Characteristics vs. Preferences in Dog Choice

Previous studies have indicated that many aspects of dog acquisition behavior, including the preferences of individuals aspiring to adopt a dog from a shelter [16], the propensity of prospective owners to undertake preparatory research [11], or where they obtain a dog from [9], may differ depending on the owners’ demographic characteristics and familial circumstances. We investigated the same characteristics in relation to the codes that they mentioned as reasons for choosing a particular dog.

We found that older owners were more likely to mention earlier experiences with similar breeds or breed types, while younger owners were more likely to mention the origin of the dog, particularly that they wanted to adopt or rescue a dog. Previous studies also showed that age was related to how owners perceive the various sources for dog acquisition. For instance, older owners were more supportive of acquiring purebred dogs compared to younger people [9], while the latter were found to prefer dog adoption over breeding [10]. Bir et al. [9] hypothesized that these differences might be due to variations in experience, as well as a higher sensitivity of younger owners to dog welfare issues. Aside from the source of the dog, younger owners were also more likely to indicate friendliness as an important factor compared to older owners, which seems to contradict what was found in theory, since older owners were more likely to indicate that their ideal dog should be socially acceptable and non-aggressive [21].

We found no association between the owners’ education level and the likelihood of mentioning any of the codes. This aligns with the findings of Diverio et al. [20], who also indicated that education level did not affect any behavioral characteristics of the ideal dog. On the other hand, King et al. [21] found that less-educated owners preferred “energetic/faithful/protective” and “socially acceptable” characteristics in their ideal dog. Moreover, Bir et al. [9] showed that people with at least a college degree were more likely to report their intention to adopt from a shelter or rescue center than those without college degrees, which was also not found in our study (although it is unclear whether or how much this reported intention would turn into action).

Our results showed that family composition indeed affected the likelihood of mentioning the majority of the codes that we extracted from the owners’ responses. The number of adults in the household was associated with a preference for trainability and choosing a dog on a whim. Owners living alone were more likely than non-single owners to adopt a dog without any self-reported expectations or reasons, similar to previous findings [41]. Adopting a dog because one likes it can lead to conflict if the animal only partially meets the expectations of several people living together [42]. Of course, it is also possible that because adults living alone may experience more loneliness [43], their efforts to alleviate loneliness may override most of their preferences, making the choice based on fewer criteria. 

Our results also showed that dog owners who lived in multi-person households were more likely to mention looking for a smart, easily trainable dog. Trainability could enable the dog to adapt and conform to the needs and expectations of a larger group [42,44]. These traits, as highlighted in previous studies [17,19], are often prioritized over impulsive behaviors among potential dog owners. Nonetheless, in households with multiple adults, the harmonious coexistence of all members may take precedence over achieving exceptional training performance [42].

Among all of the owner characteristics that we investigated, the most associations were found with whether there was a child in the household. In our sample, owners living with children were less likely to adopt a shelter or rescue dog or to choose a dog out of pity. Pets adopted from rescue organizations or found on the street often have unknown ancestry, so their possible hereditary diseases are not known. Additionally, dogs adopted from these sources can bring trauma from previous experiences [45,46], which can cause problems in a larger family later [47,48]. With young children, the potential danger—such as a resource-guarding dog [49]—can be a concern that parents may want to avoid. The age of the children can also be a determining factor for possible conflicts [50]. However, it is worth noting that animals from irresponsible breeders can also present problematic and unexpected behavioral issues due to premature weaning, inappropriate socialization, or poor genetic background [51]. Nevertheless, it is conceivable that a dog’s predictable temperament is more important for owners living with children. An alternative explanation for owners with children being less likely to adopt a shelter or rescue dog could be the lower availability of child-compatible dogs in the shelters.

In addition to avoiding shelter or rescue dogs, owners living with children in the household more often mentioned seeking dogs with “family dog” traits, especially friendly, easily manageable, and calm dogs, compared to owners living without children. This aligns with previous findings [20,21]. Although we did not ask whether the dog or the child came first in the family, it is possible that even childless individuals chose dogs with the potential future presence of children in mind. Since most dog attacks are suffered by children in the family [52], ensuring that the dog is tolerant of a child’s extreme or rough behavior can be crucial [20,21,53]. 

Parents were also more likely to emphasize the ease of training the dog, which may facilitate adaptation to a fast-paced household with a child. Regular training increases the animal’s manageability and reduces the likelihood of potential behavioral problems, leading to easier coexistence [54]. Interestingly, having a child in the household did not influence the likelihood of mentioning any type of appearance traits, including size. One might expect smaller dogs, which are becoming more popular each year, to be preferable for parents because they present a lower risk to a child and are easier to physically control [20,21,55]. However, according to dog breed stereotypes, small dogs are typically considered to be overly energetic and nervous [38], while large dogs are associated with being good “nannies” (i.e., calm and child-friendly). This discrepancy may have discouraged owners from having a preference for a specific size category.

Depending on whether a household had a dog when the newest one arrived, different preferences were found when analyzing the questionnaire. Owners who had no dogs in the household were more likely to choose the dog on a whim. This aligns with the explanation that owners who live alone do not need to consider the needs and preferences of others in the household, including other dogs. In accordance with this, owners who already had a dog were more likely to consider the next dog’s origin. Prospective owners who did not have a dog when adopting the current one were more inclined to mention traits falling under the *composite friendly/manageable* theme. Considering that the *friendly/family dog* code did not show a significant difference across owners with and without dogs, it is likely that the prospective owners were looking for calmness and manageability rather than friendliness itself. In line with this, these prospective owners were also more likely to seek an easily trainable dog. 

It is possible that the link between the number of dogs in the household and easy manageability is more indirect and mediated by other lifestyle characteristics of the owner [56]. For example, people who generally have less time for their dogs, cannot invest too much effort in dog training, or do not consider themselves to be particularly good at dog training may prefer to have only one easily manageable dog at a time. A recently acquired dog, characterized by a more amiable and easygoing temperament or displaying reduced assertiveness, as noted by Wallis et al. [57], may find smoother integration into a new household. 

In contrast, owners who already had a dog at home were more likely to consider the sport or work potential of the next dog. With an increasing number of owners embracing practical roles, such as involving their pets in sports activities as a shared hobby [58], there is a possibility that owners initiated such pursuits with their initial dog and subsequently sought a new canine companion specifically tailored for those activities. Sporting and working dogs often exhibit higher energy levels and independence compared to their regular pet counterparts, demanding more commitment from the owner [59]. This may not make them an ideal choice for a first dog, but in households with multiple dogs they can engage in activities together, helping to expend their energy [60]. It is also noteworthy that individuals with working and sporting dogs may prioritize companions with a “good drive” [30], although this trait can pose challenges to harmonious coexistence with other dogs.

Aside from household composition, we also aimed to investigate whether past experience with dogs influences prospective owners’ dog-choice behaviors. Owning dogs enhances general knowledge about dog care and fosters understanding of the owner’s unique circumstances, needs, and preferences. Moreover, experience with specific types, breeds, or bloodlines of dogs can deepen dog-specific knowledge, shaping expectations and requirements for future canine companions. Previous studies have shown that prior dog ownership affects attitudes towards dog ownership [6], potentially exerting a significant influence on breed selection decisions [61]. Contrary to some previous findings, we discovered that first-time dog owners were more likely to prioritize appearance, particularly size, when selecting their pets. This emphasis on external characteristics may stem from a lack of experience in identifying desirable behavioral traits [62]. Such novice owners may draw inspiration from social media or popular media portrayals, contributing to the emergence of trends favoring specific breeds [63]. Conversely, experienced owners may regard size as an inherent and self-evident trait, thus not explicitly mentioning it during the selection process. Among owners with prior dog-keeping experience, we observed a greater tendency to consider sport- or training-related traits. Additionally, these more experienced owners were more likely to draw upon their direct experience with specific breeds or types of dogs, consistent with findings by Menchetti [64]. 

Another characteristic that we investigated in relation to owners’ dog-choice behavior was the purpose behind keeping the dog. Our findings revealed that owners who kept dogs for sport or work were more inclined to select a breed instead of an individual dog, and to prioritize sport- or training-related characteristics encompassing both work/sport skills and intelligence/trainability. These associations are intuitively understandable and require no further elaboration. Notably, we observed no clear connection between keeping a dog solely for companionship and the qualities typically associated with a good companion dog, such as friendliness and ease of handling. One plausible explanation for this is that sporting and working dogs are valued not only for their practical roles but also for their companionship, rendering these aforementioned characteristics equally crucial in their case. Another association that we identified regarding the purpose of dog-keeping pertained to the origin of the dog. Specifically, owners who kept dogs solely for companionship were more likely to mention adopting a shelter or rescue dog or taking in a dog out of compassion. This finding aligns with previous research on shelter adoptions, where owners preferred dogs that exhibit joy in play or close proximity [17]. In contrast, intense jumping behaviors in a dog may be perceived as less desirable than a quiet and calm demeanor [19]. As previously noted, adopted dogs may exhibit less predictable behavior and, in the case of adopted puppies, may also have less predictable adult appearances in terms of size and shape. While this may pose fewer concerns for individuals seeking a pet without a specific purpose, owners engaged in dog sports tend to be more discerning about desired behaviors [65]. Additionally, cost considerations may come into play, as owners may be less willing to invest in a dog kept solely for companionship compared to a high-end sporting dog. Moreover, higher-level competitions often require pedigreed dogs, whereas most shelter and rescue dogs are mixed breeds or have unknown ancestry, limiting their potential for success in competitive events. These factors may deter owners who are interested in competitive sports or work from adopting rescue dogs.

### 4.4. Limitations

It is important to acknowledge that, like most studies on this topic, our investigation was conducted using a convenience sample, likely biased towards urban owners, young and middle-aged women, and those with a strong interest in dogs and dog ownership, while potentially under-representing owners less satisfied with their dogs. This sample composition precluded the investigation of certain demographic variables, such as the gender of the owner, which other studies have suggested to be relevant. 

Furthermore, our study was exclusively conducted on Austrian dog owners, meaning that cultural nuances in dog-keeping may have influenced our findings. For instance, we observed that working- and sporting-related characteristics were mentioned nearly twice as frequently by owners (36.86%) compared to traits like friendliness and manageability (19.31%), which may reflect specific dog-keeping habits in Austria and may not be generalizable to other countries. 

Additionally, we excluded owners who did not keep their dogs either for companionship or for working or sporting purposes, further limiting the generalizability of our results. However, compared to studies with even more specific samples (such as owners aspiring to adopt from shelters, or owners of brachycephalic dogs), our sample exhibits somewhat higher diversity. 

It is also important to note that the data were collected approximately ten years ago, and trends and preferences for dog breeds and characteristics can change over time. While we demonstrated consistency in owner preferences over time, it was for a relatively short period (8 months, on average). Following up on these changes longitudinally over several years could monitor these changes and provide stronger evidence of temporal consistency in the owners’ dog-choice behavior.

### 4.5. Avenues for Future Studies

Several intriguing research questions remain for future exploration. Firstly, we suggest collecting information on whether the current dog meets the original expectations and whether the dog has behavioral problems. Certain temperament traits, while typically neutral or positive, may function as vulnerabilities that predispose dogs to behavioral issues if not properly managed or matched with appropriate environments. These vulnerabilities may influence the compatibility between dogs and their adoptive families. For instance, prospective owners may seek a dog with high cognitive abilities, often described as ”intelligent” or “smart”, without fully understanding the corresponding need for mental stimulation, consistent training, and adequate physical exercise. If these needs are not met, the dog may develop behavioral issues such as excessive chewing, persistent barking, hyperactivity, or stereotypic behaviors, which can lead to owner dissatisfaction and potential challenges in maintaining the adoption.

It would be insightful to examine which breeds were more commonly chosen for specific characteristics (e.g., intelligence or friendliness) and whether these associations were solely based on breed stereotypes. Although our research involved many dog breeds, conducting such analyses would necessitate a larger sample or targeted data collection to achieve a more balanced sample composition. Additionally, beyond dog characteristics, it may be worthwhile to explore whether other attributes of the owners, which were not investigated in our study, influence the selection of a desired animal. Previous studies have suggested potential factors in this regard, including the owner’s gender and income [9,66], although other studies have found no significant relationship between the source of the dog and the income category [10]. It would also be an interesting direction for further research to investigate whether owners’ dog selection preferences are related to the quality of the dog–owner relationship. Finally, research has indicated that assortative mating and similarity in personality are important factors in the dog–owner relationship [28,29]. Therefore, an additional avenue for future studies could involve investigating whether the owner’s psychological characteristics, such as personality and attachment style, are linked to their preferences in choosing a dog.

## 5. Conclusions

In this study, we collected data from a large sample of Austrian pet dog owners using an open-ended question to identify the key characteristics that they considered to be important when choosing their dogs. We identified 24 distinct codes in the responses and grouped them into themes related to the dog’s appearance, origin, friendliness, training and sports skills, and demographics. Interestingly, owner-related factors like sentimental reasons and previous experience with similar breeds also emerged, which had not been highlighted in previous research. This indicates that the decision-making process for choosing a dog involves a combination of both dog- and owner-related factors.

We found that approximately 30% of the owners selected the breed instead of the individual dog, 24% chose their dogs without careful consideration, and 21.5% considered the dog’s origin. Only 20% of respondents mentioned any appearance-related traits, in contrast to behavioral traits, which were mentioned in nearly 55% of responses. The dogs’ working and sporting skills were the most frequently mentioned traits, while less than 1% of owners prioritized guarding abilities, and fewer than 2% considered basic characteristics such as sex, age, and health status as key factors in their dog selection. These findings contrast with those of previous studies that used closed-ended questions, possibly due to our study’s open-ended and retrospective design.

Characteristics related to the dog’s appearance and work/sports skills, as well as reasons like previous dog experiences and sentimental reasons, were consistent across multiple dogs. However, factors such as the dog’s friendliness, origin, and choosing the dog on a whim were less consistent. This partially supports the notion that dog-choice behaviors are a consistent characteristic of the owner and suggests that owners have consistent preferences for certain physical and behavioral traits, while spontaneous, impulsive choices are less characteristic.

We also found that variables such as the owner’s age, household composition, previous dog experience, and the intended purpose of the dog all influenced the selection process. Among all the owner characteristics, the presence of a child in the household had the most associations. Owners with children were less likely to adopt from shelters or choose a dog out of pity. For parents, concerns like resource-guarding behavior may be especially worrisome with young children.

These findings could be useful for improving dog adoption campaigns, allowing shelters to tailor their services and communication strategies to meet the preferences of owners from different demographics. Moreover, understanding the motivations and preferences behind dog acquisition can enhance both human and canine welfare and deepen our understanding of the human–dog relationship. 

## Figures and Tables

**Table 1:** Demographic characteristics of the sample.

Characteristic	Category	Count	Percentage
Gender of the owner	Male	126	11.70%
	Female	951	88.30%
Age of the owner (year)	Mean ± SD	36.59 ± 12.68	
Highest education level	Middle school	461	42.88%
	Technical school	305	28.37%
	College, university	309	28.74%
Type of residence	City (>50,000 people)	671	62.30%
	Town (2500–50,000 people)	277	25.72%
	Village (0–2500 people)	129	11.98%
Number of adults (>18 y) in the household	Single-person household	320	29.71%
	Two or more	757	70.29%
Number of children (<18 y) in the household	None	865	80.32%
	One or more	212	19.68%
Number of current dogs	Only one	574	53.30%
	Two or more	503	46.70%
Number of dogs when the current one arrived	Zero	656	61.02%
	One or more	419	38.98%
Number of dogs before the current one	None	360	33.43%
	Had dog	717	66.57%
Mixed or purebred	Mixed	344	32.00%
	Purebred	731	68.00%
Role of the dog in the family	Only for companionship	484	45.02%
	Working or sporting too	591	54.98%
Sex of the dog	Male	482	44.84%
	Female	593	55.16%
Reproductive status	Intact	487	45.30%
	Neutered or spayed	588	54.70%
Age of the dog (year)	Mean ± SD	4.14 ± 3.39	
Origin of the dog	Breeder	588	54.65%
	Private hands	265	24.63%
	Shelter or rescue dog	223	20.72%
Age at acquisition	Under 1 year old	933	86.79%
	Over 1 year old	142	13.21%

**Table 3:** Statistical details of the most parsimonious models.

Dependent: Code or Theme		Explanatory: Demographic Traits	Wald χ^2^	*p* Value	Exp(B) (95% CI)	Direction of Difference
**Appearance theme**		Previous experience with dogs	14.368	<0.001	0.556 (0.410–0.753)	No dog before > had dog before
	Size	Previous experience with dogs	13.966	<0.001	0.477 (0.323–0.703)	No dog before > had dog before
**Origin theme**		Child	6.065	0.014	0.591 (0.389–0.898)	No child > child
		N dogs when the dog arrived	4.484	0.034	1.383 (1.024–1.867)	None < had dog
		Role of the dog	7.042	0.008	0.669 (0.497–0.900)	Companion only > sport/work
		Owner age	5.032	0.025	1.014 (1.002–1.026)	Younger > older
	Shelter/rescue	Child	5.577	0.018	0.566 (0.353–0.908)	No child > child
		Role of the dog	15.175	<0.001	0.524 (0.378–0.725)	Companion only > Sport/work
		Owner age	5.812	0.016	1.017 (1.003–1.030)	Younger > older
**Friendly/** **manageable theme**		Child	6.761	0.009	1.606 (1.124–2.294)	No child < have child
		N dogs when the dog arrived	9.442	0.002	0.598 (0.431–0.830)	None > had dog
	Friendly/family dog	Child	10.678	0.001	1.917 (1. 297–2.831)	No child < child
		N dogs when the dog arrived	5.108	0.024	0.648 (0.445–0.944)	None > had dog
		Previous experience with dogs	5.156	0.023	1.581 (1.065–2.348)	No dog before < had dog before
		Owner age	5.956	0.015	1.018 (1.004–1.033)	Younger > older
**Sport theme**		Previous experience with dogs	5.819	0.016	1.418 (1.068–1.883)	No dog before < had dog before
		Role of the dog	110.933	<0.001	4. 469 (3.382–5.905)	Companion only < sport/work
	Smart/trainable	Adults	5.336	0.021	1.645 (1.078–2.511)	Single < more people
		Child	7.980	0.005	1.782 (1.193–2.660)	No child < child
		N dogs when the dog arrived	5.645	0.018	0.634 (0.436–0.923)	None > had dog
		Role of the dog	20.103	<0.001	2.391 (1.633–3.500)	Companion only < sport/work
	Work/sports skills	Child	4.437	0.035	1.497 (1.028–2.179)	No child < child
		N dogs when the dog arrived	14.789	<0.001	1.841 (1.349–2.513)	None < had dog
		Role of the dog	93.562	<0.001	7.186 (4.819–10.715)	Companion only < sport/work
Breed-based choice		Role of the dog	12.403	<0.001	1.623 (1.239–2.124)	Companion only < sport/work
Background information		Previous experience with dogs	6.056	0.014	2.039 (1.156–3.597)	No dog before < had dog before
		Owner age	6.736	0.009	0.978 (0.962–0.995)	Younger < older
Choosing on a whim		Adults	7.657	0.006	0.657 (0.487–0.885)	Single > more people
		N dogs when the dog arrived	8.882	0.003	0.634 (0.470–0.856)	None > had dog

## Data Availability

The dataset analyzed in the current study is available as Supplementary Material.

## Supplementary Materials

The following supporting information can be downloaded at: https://www.mdpi.com/article/10.3390/ani14182634/s1.
